# Case report: A relatively rare adverse event caused by carbetocin— placenta interception

**DOI:** 10.3389/fphar.2023.1112694

**Published:** 2023-03-23

**Authors:** Jun-Qiang Li, Yong-Chi Zhan, Xiao-Dong Wang

**Affiliations:** ^1^ Department of Obstetrics and Gynecology, West China Second University Hospital, Sichuan University, Chengdu, China; ^2^ Department of Obstetrics and Gynecology, The Third People’s Hospital of Chengdu, Chengdu, China

**Keywords:** placenta interception, carbetocin, cesarean section, excessive uterine contraction, case report

## Abstract

Placenta interception is extremely rare, and herein, we report the case of a 33-year-old woman with placenta interception during cesarean section caused by carbetocin, which was intravenously injected immediately after delivery of the infant to prevent postpartum hemorrhage. There was no sign of placental separation in the third stage of labor. A protuberance with gross subserous blood vessels in the left fundus of the uterus was detected and was misdiagnosed as placental accreta. The patient was transferred to the district referral hospital to manage the protuberance after stitching up the incision. On opening the original incision in the tertiary hospital, the protuberance disappeared, and the placenta was found in the lower segment of the uterus cavity. The intercepted placenta showed a spontaneous resolution from the uterine wall and was removed intact. This case report details the typical presentation of placenta interception to make obstetricians aware of the possibility that carbetocin might lead to this adverse event.

## Introduction

Carbetocin is an artificial synthetic long-acting oxytocin that can cause uterine rhythmic contraction, which enhances the frequency, intensity, and duration of the uterine contraction. Its effect is observed 2 min after intravenous injection and can last for 60 min (Ai et al., 2021). Like other effective uterotonics, carbetocin is recommended for the prevention of postpartum hemorrhage (PPH) for cesarean section by the World Health Organization (WHO) (WHO Recommendations, 2018). Similar to oxytocin, it has fewer adverse effects and high efficacy in preventing PPH during cesarean section after intravenous injection (Taheripanah et al., 2018; Onwochei et al., 2020). In addition, carbetocin has heat-stable formulation compared to oxytocin (Malm et al., 2018; WHO Recommendations, 2018). Therefore, it may be applied extensively as an alternative to oxytocin as a primary preventive treatment of PPH in cesarean section (Theunissen et al., 2018), especially in low and middle-income countries due to inadequate cold-chain transport and storage conditions (Torloni et al., 2016).

Herein, we present a case report of placenta interception caused by carbetocin during a cesarean section. To our knowledge, this is the first reported case of an adverse event caused by carbetocin and the first described case of placenta interception during cesarean section.

## Case presentation

**FIGURE 1:**
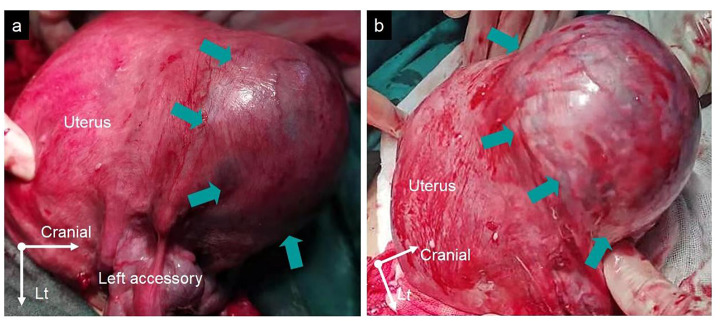
**(A,B)** The protuberance with gross subserous blood vessels in the left fundus of the uterus from different perspectives measuring ∼10 × 10 × 8 cm (green arrows).

**FIGURE 2:**
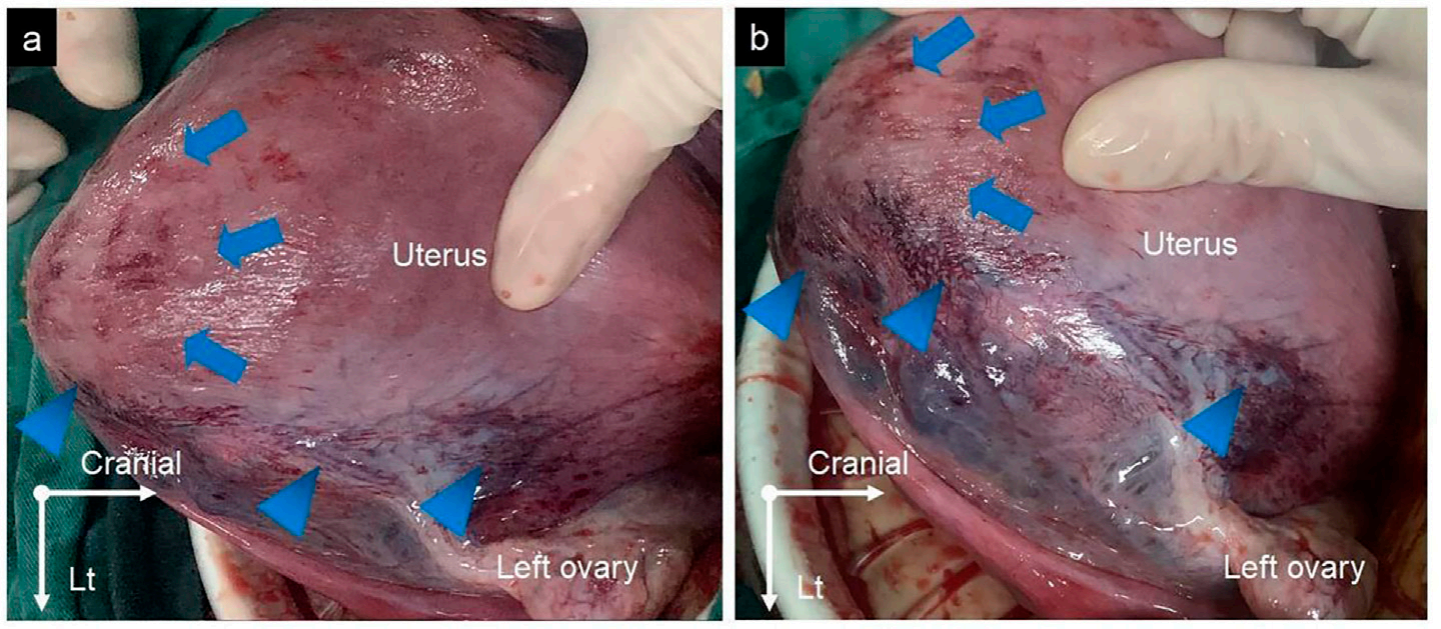
**(A,B)** The protuberance in the left fundus of the uterus disappeared (blue arrows), with a bruise on the left uterine horn (blue arrowheads).

A 33-year-old woman, gravida 4 para 1, underwent a cesarean section under epidural anesthesia with ropivacaine for premature rupture of membranes and fetal breech presentation at 34 weeks gestation at a local hospital. After the cesarean delivery of the infant and before the expulsion of the placenta, 100 μg of carbetocin was intravenously injected over 1 min to prevent PPH. After a few minutes, there was no sign of placental separation, and a protuberance (∼10 × 10 × 8 cm) with gross subserous blood vessels was detected in the left fundus of the uterus (Figure 1), with a quarter of the placenta retained in the uterine cavity without active bleeding. The protuberance, which contained the other three-quarters of the placenta, appeared oval in shape, smooth surface, medium texture, and consistent with the color of the uterus. The operator of the local hospital considered that the protuberance was caused by placenta accreta and they could not handle it due to insufficient blood supply. So the umbilical cord was ligated, and the uterus and abdominal incision sutured before the patient was transferred to the district referral hospital, a tertiary hospital in Chengdu. The patient was rehydrated and prepared for an emergency uterine wedge resection after sufficient blood was prepared. The laparotomy revealed that the protuberance in the left fundus of the uterus had disappeared, and the uterine morphology seemed to be normal, with a bruised left uterine horn (Figure 2). Opening the original uterine incision, the complete placenta was found in the lower segment of the uterus cavity, so the retained placenta was removed intact and the incision was stitched up. The duration between the two interventions, from the end of the first operation to the beginning of the second, was 109 min and the total blood loss, from the beginning of the first operation to the end of the second, was 450 mL. Finally, the patient recovered well, and the pathologic examination of the placenta excluded the diagnosis of placenta accreta.

## Discussion

The WHO recommends active management of the third stage of labor to prevent PPH through the administration of a uterotonic immediately after the birth of an infant (WHO Recommendations, 2018). However, using uterotonic in the third stage of labor can lead to excessive uterine contraction (EUC) in some cases. The most common result of EUC in the third stage of labor is placenta incarceration which forms a constriction ring in the uterus, thereby retaining the detached placenta in the uterine cavity. Placenta interception, which is first mentioned in the Chinese instructions of carbetocin (2009 edition) of Huiling Pharmaceutical Company, is also considered a rare outcome of EUC in the third stage of labor and is described as the partial or complete embedment of the placenta in the uterine wall, forming an oval, smooth, medium-textured protuberance consistent with the color of the uterine surface.

Placenta interception during a cesarean section due to EUC caused by carbetocin is extremely rare and has not been previously reported. There are no established guidelines for the prevention and management of placenta interception. In the present case, the reason for placenta interception is regarded as the result of EUC due to hypersensitivity to carbetocin. The duration between the two interventions was 109 min, which was longer than the action time of carbetocin, so the intercepted placenta showed a spontaneous resolution from the uterine wall once the carbetocin action time elapsed. According to the management of dealing with EUC, administering a tocolytic agent, such as Magnesium sulfate, or terbutaline, may assist in separating the intercepted placenta from the uterine wall before the carbetocin action time elapsed (Simpson and Miller, 2011).

## Conclusion

Our case shows that carbetocin might lead to placenta interception and provides the typical presentation of placenta interception to familiarize obstetricians with this adverse event.

## Data Availability

The original contributions presented in the study are included in the article/Supplementary Material, further inquiries can be directed to the corresponding author.
