# Corrigendum: Case report: A relatively rare adverse event caused by carbetocin—placenta interception

**DOI:** 10.3389/fphar.2024.1520266

**Published:** 2024-11-11

**Authors:** Jun-Qiang Li, Yong-Chi Zhan, Xiao-Dong Wang

**Affiliations:** ^1^ Department of Obstetrics and Gynecology, West China Second University Hospital, Sichuan University, Chengdu, China; ^2^ Department of Obstetrics and Gynecology, The Third People’s Hospital of Chengdu, Chengdu, China

**Keywords:** placenta interception, carbetocin, cesarean section, excessive uterine contraction, case report

In the published article, there was an error in the **Funding** statement. One of the funding fund projects was omitted. The correct **Funding** statement appears below.

